# Improved coverage of cDNA-AFLP by sequential digestion of immobilized cDNA

**DOI:** 10.1186/1471-2164-9-480

**Published:** 2008-10-13

**Authors:** Arne Weiberg, Dirk Pöhler, Burkhard Morgenstern, Petr Karlovsky

**Affiliations:** 1 Molecular Phytopathology and Mycotoxin Research Division, University of Goettingen, Grisebachstrasse 6, 37077 Goettingen, Germany; 2 Department for Bioinformatics, University of Goettingen, Goldschmidtstrasse 1, 37077 Goettingen, Germany

## Abstract

**Background:**

cDNA-AFLP is a transcriptomics technique which does not require prior sequence information and can therefore be used as a gene discovery tool. The method is based on selective amplification of cDNA fragments generated by restriction endonucleases, electrophoretic separation of the products and comparison of the band patterns between treated samples and controls. Unequal distribution of restriction sites used to generate cDNA fragments negatively affects the performance of cDNA-AFLP. Some transcripts are represented by more than one fragment while other escape detection, causing redundancy and reducing the coverage of the analysis, respectively.

**Results:**

With the goal of improving the coverage of cDNA-AFLP without increasing its redundancy, we designed a modified cDNA-AFLP protocol. Immobilized cDNA is sequentially digested with several restriction endonucleases and the released DNA fragments are collected in mutually exclusive pools. To investigate the performance of the protocol, software tool MECS (Multiple Enzyme cDNA-AFLP Simulation) was written in Perl. cDNA-AFLP protocols described in the literatur and the new sequential digestion protocol were simulated on sets of cDNA sequences from mouse, human and *Arabidopsis thaliana*. The redundancy and coverage, the total number of PCR reactions, and the average fragment length were calculated for each protocol and cDNA set.

**Conclusion:**

Simulation revealed that sequential digestion of immobilized cDNA followed by the partitioning of released fragments into mutually exclusive pools outperformed other cDNA-AFLP protocols in terms of coverage, redundancy, fragment length, and the total number of PCRs. Primers generating 30 to 70 amplicons per PCR provided the highest fraction of electrophoretically distinguishable fragments suitable for normalization. For *A. thaliana*, human and mice transcriptome, the use of two marking enzymes and three sequentially applied releasing enzymes for each of the marking enzymes is recommended.

## Background

Transcriptome analysis is vital to all fields of biology concerned with spatial and temporal patterns of gene activity. Hybridization of labeled cDNA to oligonucleotides immobilized in two-dimensional arrays became the method of choice for fast access to the transcriptome of model organisms. A disadvantage of DNA microarrays is that they belong to closed-end methods, which only work with known genes. A growing need for open-end transcriptomics and transcriptome analysis-based gene discovery tools inspired the development of transcript analysis techniques relying on the electrophoretic separation of amplified cDNA fragments.

Two major strategies dominate cDNA fragment pattern-based transcriptomics. PCR primed by oligo(dT) in conjunction with short, random primers annealing at a very low temperature is the basis of cDNA Differential Display invented by Liang and Pardee [1], while digestion of cDNA with restriction endonucleases followed by the attachment of double-stranded adapters and specific amplification of subsets of these fragments, originally developed for genome fingerprinting [2], is used in cDNA-AFLP (Amplified Fragment Length Polymorphism of cDNA) [3-6]. The latter method gained popularity after radioactive labels attached to primers [2,3,5] or incorporated into the product as phosphorylated nucleotides [7] were replaced by fluorescent dies [4,6]. Software that facilitates the analysis of a large number of cDNA-AFLP electropherograms was developed available (e.g., [8]). Because fragments serving as PCR templates are terminated by adapters that provide specific binding sites for primers and because the amplification takes place under stringent conditions, mispriming is limited. The higher reproducibility of cDNA-AFLP vs. cDNA Differential Display is accompanied by a higher complexity of experimental protocol. Both cDNA Differential Display and cDNA-AFLP are often used as gene discovery tools because fragments of interest can be extracted from the electrophoretic matrix and sequenced.

Detection of a particular transcript by cDNA-AFLP depends on the presence of recognition sites for restriction endonucleases in the complementary DNA sequence. On the other hand, the same transcript may generate several cDNA-AFLP signals when more than one several enzyme combinations are used. As the cost of the experiment depends on the number of primer combinations used, it is desirable to optimize the method for minimal redundancy and to minimize the number of PCR reactions. In addition, it is desirable to maximize fragment length in order to obtain more informative sequences. To this end, we suggest a modified cDNA-AFLP protocol based on sequential digestion of cDNA immobilized on a solid matrix, followed by the partitioning of the released fragments into mutually exclusive pools that serve as template for PCR.

The performance of different cDNA-AFLP protocols can be compared by computer simulations. Several programs are available for the simulation of cDNA-AFLP. GenEST [9,10] links sequence information to cDNA-AFLP patterns, predicting fragments generated from known transcripts and identifying transcripts that match experimentally detected fragments. Kivioja's software [11], AFLPinSilico [12,13], InSilico AFLP [14,15], InSilico Simulation [16,17], and the commercial software ReComb (Keygene, Wageningen, Netherlands) simulate cDNA-AFLP analysis for one enzyme pair and a set of cDNA sequences given as input. Kivioja's software also optimizes the number of selective nucleotides in PCR primers, which affects both the quality of band patterns and the number of PCRs, reducing the experimental effort by 25–50%. AFLPinSilico was used to compare the efficiency of enzyme pairs and their combinations with respect to transcript coverage, fragment size, and the proportion of 3'-untranslated regions [5,18]. Breyne et al. [5] calculated that a maximum coverage of 60% could be achieved for 5000 full-length cDNA sequences from *Arabidopsis thaliana *for a single enzyme pair. The use of a second enzyme pair increased the coverage to over 80%, but the redundancy was not determined.

Neither of the tools listed above is suitable for simulating the modified cDNA-AFLP protocol described in this work. We therefore developed a tool called MECS (Multiple Enzyme cDNA-AFLP Simulation) and used it to optimize the number and order of restriction enzymes in our protocol and to compare the protocol with other cDNA-AFLP systems in terms of coverage, redundancy, and experimental effort. We used sets of cDNA sequences from *Arabidopsis thaliana*, mouse, and human.

## Results and discussion

### New cDNA-AFLP protocol based on sequential digestion of immobilized cDNA

**Figure 1:**
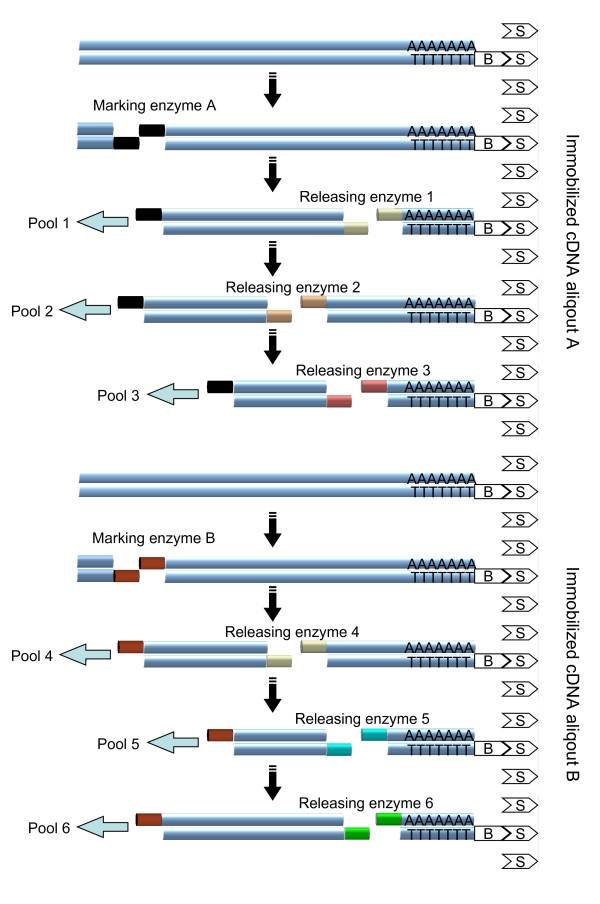
**Protocol for sequential digestion cDNA-AFLP**. Biotinylated cDNA molecules are bound to streptavidin matrix. Each combination of a marking enzyme (A and B) with a releasing enzyme (one to six) generates a cDNA fragment pool.

Our improvement of the cDNA-AFLP protocol, based on a multiple restriction digestion applied sequentially on immobilized cDNA, is depicted in Fig. 1. The procedure starts with binding anchored oligo(dT) primers to a column. We use biotinylated primer of the sequence Biotin-GAGAGAGCGGCCGCGAGAGATTTTTTTTTTTTTTTTTTTTV and reaction tubes coated with streptavidin, but any immobilized oligo(dT) nucleotide should work. mRNA is hybridized to the primer, followed by a washing step that removes RNA species not possessing poly(A)-sequences. Double-stranded cDNA is synthesized using established protocols while the immobilized oligonucleotide serves as primer for the first strand. cDNA immobilized on the column is digested with the first restriction enzyme (called "marking enzyme A"), and released fragments are removed by washing [19]. This step eliminates redundancy within fragment pools originating from the same column [5]. A second digestion is performed with another restriction enzyme ("releasing enzyme 1"), and the DNA fragments are collected for amplification as pool 1 (Fig. 1A, step 2). The fraction of transcripts covered by this pool corresponds to the coverage of classical cDNA-AFLP protocols [3]. A third restriction enzyme ("releasing enzyme 2") is applied, generating pool 2 (Fig. 1A, step 3). The consecutive application of several releasing enzymes to immobilized cDNA improves the coverage, because fragments lacking recognition sequence for the first releasing enzyme may be cleaved by one of the following enzymes. The redundancy among pools originating from the column remains zero, because the first releasing enzyme that digests the fragment removes the end generated by the marking enzyme, which is necessary for adapter ligation and amplification [2]. The following releasing enzymes might digest cDNA left on the column, but the released fragments will not be amplified. As a result, we amplify only fragments delimited by cleavage sites for the marking enzyme at the 5' end with respect to the original mRNA and one of the releasing enzymes at the 3' end. For each cDNA, at most one such fragment is amplified.

cDNA fragments lacking recognition site for marking enzyme A escape detection in fragment pools originating from the first column. We therefore extended the protocol by immobilizing a second aliquot of mRNA on another column and repeating the procedure with a different marking enzyme designated B. This strategy improves coverage but it also causes redundancy, unless compensatory measures are taken (see below). Transcripts with recognition sites for both marking enzymes and for at least one of the releasing enzymes applied to each column will be represented in two pools.

Theoretically, redundancy could be completely eliminated and coverage improved by implementing additional steps. In principle, concomitant digestion of cDNA immobilized on all columns with all marking enzymes would eliminate redundancy completely. The reason is that a cDNA molecule containing recognition sequences for several marking enzymes would be "visualized" only by the enzyme that cleaves closest to its 3' end. Fragments of this cDNA would not appear in the pools from the other marking enzymes. If all marking enzymes are applied to all columns, the choice of the marking enzyme recognition sequence (DNA ends) used for the ligation is determined merely by the choice of the adapters. If the set of the releasing enzymes is identical for all marking enzymes, one column can be used for all digestions. A drawback of this strategy is that the average size of cDNA-AFLP fragments will be reduced, generating less informative sequences. For example, a concomitant digestion with two marking enzymes that cleave with frequencies f_1 _and f_2 _will generate immobilized fragments of an average length

L_conc _= 1/[2*(f_1 _+ f_2_)],

while separate digestion on two columns will lead to immobilized fragments of the average length

L_sep _= (f_1 _+ f_2_)/[4 * f_1 _* f_2_].

(The estimates hold under the assumption that cDNA molecules are much longer than the average length of fragments produced by both enzymes.) The reduction of the length of the immobilized cDNA fragments generated by marking enzymes applied concomitantly increases the chance that a fragment will not be cleaved by any releasing enzyme and will therefore escape detection. On the other hand, the same cDNA molecule might be detected twice when marking enzymes are applied separately. This redundancy might be useful if the sequence of the longer fragment is informative while the sequence of the shorter fragment is entirely non-coding. If both marking enzymes are applied concomitantly, only the shorter fragment can be detected. An optimal strategy, which will be a compromise between redundancy suppression and fragment length maximization, can be found by simulation if the cDNA sequences are known.

While a pre-digestion of immobilized DNA with all marking enzymes is sufficient to completely eliminate redundancy, it impairs the coverage. After the digestion with marking enzymes, cDNA sequences that remain on the column and that lack recognition sites for any releasing enzyme will escape detection. The higher the number of marking enzymes concomitantly used, the shorter will be the fragments left on the column, and the more of them will be depleted of sites for the releasing enzymes. Sequences bound on the column that are terminated by sites for marking enzymes but that do not possess recognition sites for releasing enzymes can theoretically be recovered. To this end, adapters can be ligated to on-column bound DNA after the last releasing enzyme treatment, and fragments can be amplified with one primer complementary to the adapter and the other primer complementary to the signature sequence incorporated into the 3' terminus of cDNA via oligo(dT) primer. (For example, primer GAGAGAGCGGCCGCGAGAGA would be suitable for the biotin-labeled poly(dT)-oligonucleotide used in our work; see above.) Therefore, the extension is not included in cDNA-AFLP simulations described below.

### Optimization of the number of marking enzymes

The purpose of using multiple marking and releasing enzymes in our modification of the cDNA-AFLP protocol was to improve the coverage. The effect of the number of marking enzymes on coverage was investigated by computer simulations. High-quality cDNA sequence data from NCBI Reference Sequence collection for human, *Arabidopsis thaliana*, and mouse were used (Tab. 1). For the selection of marking and releasing enzymes, a list of target sequences of 18 enzymes (Tab. 2) with four- and five-nucleotides recognition sequences was used as input for software tool MECS (see below). The number of marking enzymes ranged from one to four while two releasing enzymes were used in all simulations.

**Table 1:** EST sequence datasets

**Organism**	**EST database**	**FASTA file size (MB)**	**No. ESTs**	**Nucleotide letters**	**Ø EST length (nucleotides)**	**Ambiguous bases/1000 nucleotides**
***Arabidopsis thaliana***	Complete UniGene set	48.1	29215	42.9 E06	1467	0.9 E-01
	
	NM_RefSeq sequences	28.9	16710	26.8 E06	1606	2.6 E-04

						

***Mus musculus***	Complete UniGene set	108.0	66691	93.9 E06	1439	0.94
	
	NM_RefSeq sequences	14.4	4044	14.1 E06	3067	1.3 E-02

						

***Homo sapiens***	Complete UniGene set	131.1	85967	114.5 E06	1335	0.73
	
	NM_RefSeq sequences	24.9	6537	24.5 E06	3746	0.4 E-02

UniGene ESTs of Arabidopsis Build #58, mouse Build #162 and human Build #201 all from the NCBI library collection were chosen as test sequences and used for the simulation of different cDNA-AFLP protocols. RefSeq sequences with a _NM identifier provided a subset of high quality ESTs in terms of length and undefined nucleotides.

**Table 2:** Restriction enzymes used for simulations

	**Restriction enzyme**	**Recognition site**	**Occurrence in RefSeq EST sets (%)**		
			
			*Arabidopsis thaliana*	*Homo sapiens*	*Mus musculus*
1	MboI	▼GATC	97.2	98.8	82.5
			
2	HpaII	C▼CGG	84.4	93.3	64.7
			
3	HinP1I	G▼CGC	60.3	89.4	61.7
			
4	Csp6I	G▼TAC	87.9	97.5	81.4
			
5	TaqI	T▼CGA	95.3	89.4	64.2
			
6	TasI	▼AATT	97.5	98.2	80.2
			
7	MseI	T▼TAA	96.3	97.2	78.9
			
8	FatI	▼CATG	96.4	99.7	83.5
			
9	MaeI	C▼TAG	88.6	95.1	76.3
			
10	MaeII	A▼CGT	86.7	92.0	64.8
			
11	ApoI	R▼AATTY	82.7	86.2	68.5
			
12	BstYI	R▼GATCY	80.0	90.5	70.4
			
13	AcyI	GR▼CGYC	37.8	70.3	46.4
			
14	BmeT110I	CY▼CGRG	49.6	79.3	52.8
			
15	Cfr10I	R▼CCGGY	49.6	68.6	45.1
			
16	CfrI	Y▼GGCCR	45.3	90.3	68.7
			
17	BsaWI	W▼CCGGW	64.1	60.1	44.2
			
18	TatI	W▼GTACW	63.5	86.8	72.7

All these restriction enzymes are commercially available, supplying companies can be found on the REBASE [23] homepage. Frequency of occurrence in EST sets were calculated using NCBI Reference Sequence collections without repetition, sequences containing more than one copy of a recognition site counted only once.

**Figure 2:**
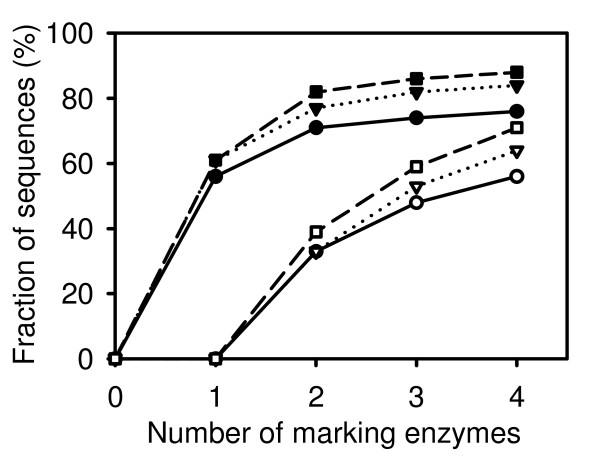
**Effect of the number of marking enzymes on coverage and redundancy**. For each of the RefSeq EST set (Tab. 1), enzyme combinations leading to the best coverage in sequence digestion protocol with two releasing enzymes were used for simulation with MECS. Coverage (filled symbols) and redundancy (open symbols) were plotted against the number of marking enzymes. Triangles connected with dashed lines: mouse; squares connected with dotted lines: human; circles connected with full line: *Arabidopsis*.

In the first part of the calculation, MECS determined the fraction of sequences in which the recognition sequence(s) for at least one marking enzyme occurred at a distance of at least 40 bp from the 3' end of the cDNA. The marking enzyme with the highest occurrence in this set was then used for simulations. When several marking enzymes occurred with the same frequency, the enzyme(s) that digested the largest fraction of sequences more than once was selected as marking enzyme(s). Using this marking enzyme(s), MECS calculated the coverage and redundancy of cDNA-AFLP for all combinations of two releasing enzymes taken from the list in Tab. 2. Coverage and redundancy for the releasing enzyme combination with the highest coverage (and lowest redundancy when several combinations provided equal coverage) are shown in Fig. 2. Increasing the number of marking enzymes from one to four improved the coverage, but the improvement per added enzyme declined with the number of marking enzymes. At the same time increasing the number of marking enzymes dramatically increased redundancy (Fig. 2).

Depending on the demands for coverage and the acceptable level of redundancy, choosing one to three marking enzymes appears a reasonable compromise. It must be pointed out that the coverage values in Fig. 2 were obtained for the most suitable combinations of marking and releasing enzymes selected from a set of 18 restriction endonucleases. If such an optimization is not possible because sequence data are not available, a lower coverage should be expected. For example, the coverage was as low as 12% (one marking enzyme), 32% (two marking enzymes), and 45% (three marking enzymes) for the worst enzyme combinations for the mouse cDNA set.

### Comparison of cDNA-AFLP protocols

Five cDNA-AFLP protocols were compared for each cDNA sequence collection: classical protocols with one and two enzyme pairs (coverage was calculated using a merge of both fragment sets in the latter case); sequential digestion protocol with two and three releasing enzymes; and a "flip-flop" strategy, in which marking and releasing enzymes swap their roles. This strategy was first described by Fukumura et al. [18] as an improvement of cDNA-AFLP protocol that eliminates redundancy.

The results of the comparison are shown in Tab. 3. Concerning the coverage, the sequential digestion protocol was superior. Comparing the best enzyme combinations, sequential digestion provided a coverage 6–11% (two releasing enzymes) or 9–13% (three releasing enzymes) better than the next best protocol. The redundancy of the sequential digestion was comparable with that of the classical protocol with two enzymes for human and mouse data but was markedly less than redundancy of the classical protocol for *Arabidopsis *data. cDNA fragments generated with the sequential protocol were longer than fragments resulting from the classical protocol with two enzymes for human and mouse data, but shorter for *Arabidopsis *data. The flip-flop strategy generated the shortest fragments in all three data sets.

**Table 3:** Comparison of cDNA-AFLP protocols

**Origin of EST**	**Protocol**	**Marking enzyme**	**Releasing enzyme**	**Sequence coverage**	**Sequence redundancy**	**Fragment length (nucleotides)**
***Arabidopsis thaliana***	Single pair of enzymes	TaqI	TasI	49 %	0 %	209
	
	Double pair of enzymes	FatI	TasI	65 %	29 %	203
					
		MboI	MseI			
	
	Sequential digestion	FatI (A)	MboI (1), TasI (2)	75 %	25 %	179
					
		MboI (B)	FatI (3), TasI (4)			
		
		FatI (A)	MboI (1), MseI (2), TasI (3)	78 %	30 %	183
					
		MboI (B)	FatI (4), MseI (5), TasI (6)			
	
	Flip/Flop	FatI	MboI	64 %	0 %	155
					
		MboI	FatI			

**Mouse**	Single pair of enzymes	MboI	MseI	54 %	0 %	230
	
	Double pair of enzymes	MboI	TasI	75 %	32 %	230
					
		Csp6I	TaqI			
	
	Sequential digestion	MboI (A)	Csp6I (1), FatI (2)	85 %	35 %	258
					
		Csp6I (B)	MboI (3), FatI (4)			
		
		MboI (A)	Csp6I (1), MseI (2), FatI (3)	87 %	41 %	270
					
		Csp6I (B)	MseI (4), MboI (5), FatI (6)			
	
	Flip/Flop	MboI	Csp6I	75 %	0 %	211
					
		Csp6I	MboI			

**Human**	Single pair of enzymes	HinPI	MseI	61 %	0 %	260
	
	Double pair of enzymes	MboI	MseI	76 %	33 %	223
					
		Csp6I	TaqI			
	
	Sequential digestion	MboI (A)	FatI (1), Csp6I (2)	82 %	31 %	249
					
		Csp6I (B)	MboI (3), FatI (4)			
	
		MboI (A)	Csp6I (1), HinPI (2), FatI (3)	85 %	35 %	274
					
		Csp6I (B)	MboI (4), FatI (5), HinPI (6)			
	
	Flip/Flop	MboI	Csp6I	76 %	0 %	228
					
		Csp6I	MboI			

cDNA-AFLP protocols were simulated with MECS software on *Arabidopsis*, mouse and human RefSeq ESTs. For the sequential digestion strategy, the order of marking enzymes (A, B) and releasing enzymes (1 to 6) is specified (see Fig. 1 for details).

Increasing the number of releasing enzymes from two to three in the sequential digestion only marginally improved performance and is therefore not recommended. It appears that the classical protocols should be abandoned: the one-enzyme variant has inferior coverage, and the use of two enzymes improved the coverage to the same level as the flip-flop protocol, but in contrast to the latter incurred a relatively high redundancy. The flip-flop protocol generated shorter fragments and provided lower coverage than the sequential digestion protocol with two releasing enzymes, but it completely eliminated redundancy, significantly reducing the effort for the amplification and separation. The experimenter may chose between flip-flop and sequential digestion depending on his/her demands and resources. It is important to note that ranking of cDNA-AFLP protocols by performance differed with the source of sequence data. This may reflect species-specific differences in the frequency of relevant recognition sequences for restriction enzymes, but the quality of sequence data also affects protocol performance (see section 6).

A widely used cDNA-AFLP protocol is the one published by Breyne et al. [5]. It is based on cDNA immobilized on magnetic beads and uses BstYI and MseI as the marking and releasing enzymes, respectively. Our simulations of this protocol on *A. thaliana*, human, and mouse EST sets predicted a coverage of 49, 56, and 51%, respectively. Breyne et al. [5] simulated the protocol on 5000 *A. thaliana *ESTs and calculated a coverage of 60%. Using the sequential digestion protocol with the best combinations of enzymes listed in Tab. 3 resulted in coverage of 78% for *A. thaliana*, 85% for human, and 87% for mouse.

### Minimizing experimental effort

#### Fragment pool size, the number of PCR reactions, and the proportion of analyzable fragments

A cDNA-AFLP fragment pool is the set of fragments washed from the column after treatment with a releasing enzyme and ligated to compatible adapters. The number of fragments in a pool is usually much larger than can be resolved by electrophoresis. A central principle of AFLP is to partition these fragments into subsets by PCR, using primers consisting of a sequence complementary to the adapters with N additional nucleotides attached to the 3' end (so called selective nucleotides [2]). All 4^N ^combinations of N selective nucleotides must be used for each fragment pool to visualize all fragments that may occur in the pool. In this way, PCR divides fragment pools into 4^N ^sets. Each additional nucleotide at the 3' end of the primer reduces the number of amplified fragments approximately four times while multiplying the number of PCR reactions by four. The total number of PCR reactions required for the analysis of fragment pools determines the experimental effort.

Fragments of the same size co-migrate during electrophoresis. A change in the intensity of a fragment within a group of co-migrating bands may remain undiscovered, particularly when the fragment is derived from a scarce transcript. The probability of co-migration grows with the number of fragments in a PCR reaction. On the other hand, using too many selective nucleotides increases the number of PCR reactions. Moreover, it may impair the normalization of signal intensities, which is required when cDNA-AFLP profiles are analyzed quantitatively. The normalization algorithm we use is based on trimmed means rather than on the total or average intensities, because fragments of transcripts affected by the treatment have to be excluded from the calculation of a normalization factor [20]. The algorithm requires that a minimal number of analyzable fragments are present in an electrophoresis lane. We set this number to 20 and excluded patterns (products resulting from a single PCR reaction) consisting of fewer then 20 fragments.

#### Estimating the optimal number of selective nucleotides for PCR primers

**Figure 3:**
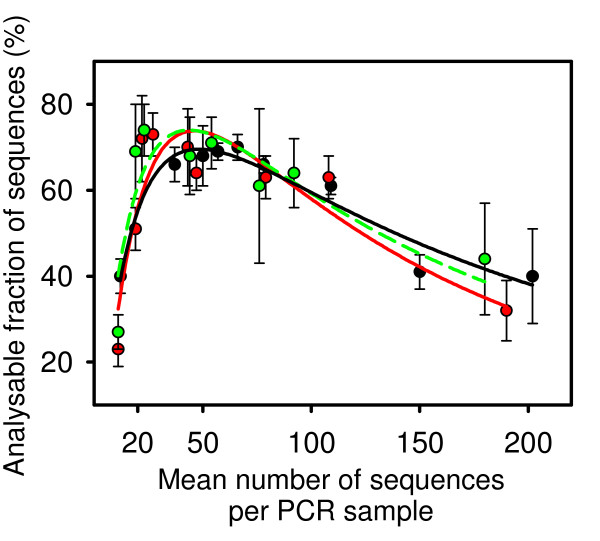
**Effect of the number of PCR products on the fraction of analyzable fragments**. For RefSeq EST data sets (Tab. 1), randomly selected enzyme combinations were used to simulate cDNA-AFLP with the sequential digestion. One to three selective nucleotides were attached to PCR primers. For each PCR reaction, the fraction of analyzable fragments (fragments between 40 bp and 700 bp with a length that occurred only once, at least 20 products per PCR) was plotted against the number of PCR products. Vertical bars indicate standard error. Black: *Arabidopsis*; red: mouse; green: human.

To determine the optimal number of selective nucleotides N, we need to know the size of the fragment pool that will be partitioned by PCR and the optimal number of products per PCR reaction. The probability of co-migration during electrophoresis increases with decreasing N, while the probability that a PCR reaction will generate fewer than 20 products grows with N. To determine the optimal number of PCR reactions, we simulated cDNA-AFLP with the sequential digestion protocol for two marking and two to three releasing enzymes. Enzyme combinations were randomly selected from Tab. 2. PCR was simulated with primers containing one to three selective nucleotides (total for both primers). For each PCR reaction, the products were sorted by size, and fragments of any size that occurred more than once were eliminated. Furthermore, products shorter than 40 bp or larger than 700 bp were eliminated. The fraction of fragments remaining in the set after the treatment was scored "analyzable." The results of these simulations are summarized in Fig. 3. Even with the optimal number of PCR products, only about 75% of the fragments are analyzable. Therefore, coverage values predicted by simulations that do not take co-migration into account and do not eliminate fragments that are too short or too long have to be reduced accordingly.

The source of EST data has no effect on the fraction of analyzable fragments. The optimal number of PCR products lies between 25 and 70. We must remember, however, that these fragments will be randomly distributed among PCR reactions. An N that leads to an average number of PCR products close to 25 would lead to a significant number of PCRs with fewer than 20 products, and such PCRs cannot be used for quantitative analysis. According to Fig. 3, N should be chosen to maximize the number of PCR reactions with the expected number of products between 25 and 70. We recommend that researchers select an N that will on average result in 30 to 70 PCR products. Because the number of PCR reactions for N selective nucleotides is 4^N^, the optimal value of N can be determined from the following inequities:

M/30 > 4^N ^> M/70

where M is the number of fragments in a pool. This leads to

logM/0.602 - 2.45 > N > logM/0.602 - 3.06.

Because (3.06 - 2.45) > 1, for certain M values no integer N satisfying both inequalities exists. In such a case, the closest value below the recommended range should be chosen. For example, 5000 fragments in a pool lead to recommendations N < 3.48 and N > 3.08. When 3 (as the closest value below the range) is used, the average number of PCR products in a pool will be 5000/64 = 78.1, which is reasonably close to the optimum in Fig. 3. Choosing N = 4, which would lead to an average number of fragments in a pool 5000/256, would waste half of the experimental effort, because the number of products in about 50% of PCR reactions would drop below 20, which is the limit set for normalization.

#### The effect of partitioning cDNA-AFLP fragments into pools on the total number of PCR reactions

The number of selective nucleotides N and consequently the number of PCR reactions 4^N ^is chosen based on the number of fragments in a pool as described in the previous section. In classical protocols, the template is digested with a pair of enzymes to generate a single fragment pool. When different enzyme combinations are used to enhance the coverage, several independent fragment pools are generated. In multiple digestion protocols, the order in which releasing enzymes are applied affects the partitioning of fragments into pools.

Let the number of PCR reactions required to analyze a pool of M fragments be 4^N^. Let the fragments be partitioned into P equally large pools and the number of PCR reactions required per pool according to the rules set in the previous section be 4^Q^. If M/P is divisible by 4^n ^for integer n, Q equals N - ln_4_(M/P) and the total number of PCR reactions does not change after partitioning fragments into pools: 4^N ^= P*4^Q^. When M/P is not dividable by 4^n^, the total number of PCR reactions for fragments partitioned into pools may be lower or equal to the number of PCR reactions that would be required if all fragments were in one pool: 4^N ^≥ P*4^Q^. Sequential digestion protocols may thus reduce the total PCR effort.

**Figure 4:**
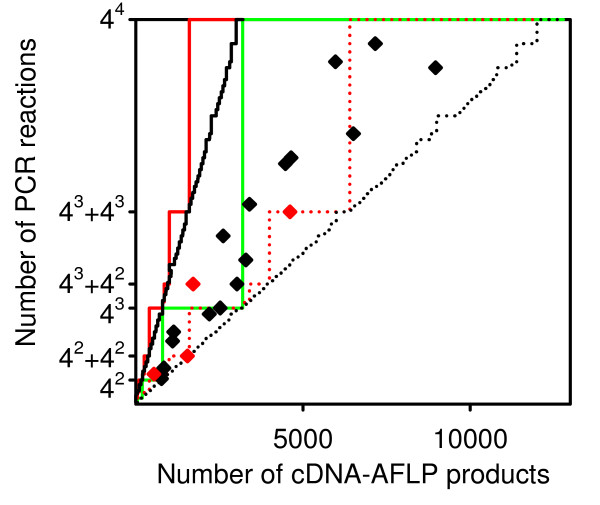
**Effect of partitioning fragments into mutually exclusive pools on the total number of PCRs**. The lowest number of PCR reactions generating not more than 50 products per PCR on the average is plotted against the number of cDNA-AFLP fragments. PCR is performed either on all fragments in one pool (green line, the number of PCRs equals to the number of primer combinations) or on two (red lines) or six (black lines) mutually exclusive pools of fragments (PCRs are summed up over all pools). For two and six fragment pools (red and black lines, respectively), dotted lines depict the optimal partition of fragments (minimum number of PCRs) while full lines depict the least favorable partition of fragments (maximum number of PCRs). The results of simulations are shown as diamonds.

Fig. 4 displays the relationship between the number of fragments and the total number of PCR reactions for one, two, and six fragment pools. To simplify the calculation, a threshold value of 50 fragments was chosen, and the lowest number of PCR reactions expected to generate fewer than 50 products per PCR on the average was chosen for each pool. For protocols with two and six fragment pools, the number of PCR reactions was summed over all pools for two kinds of partitioning of the fragments into pools: the most favorable partitioning, leading to the lowest total number of PCR reactions (dotted lines), and the most unfavorable partitioning, leading to the highest number of PCR reactions (continuous line). Data points between these two extremes represent the results of simulations on EST sequences described in the previous sections. These results confirmed that partitioning fragments into pools may reduce the total PCR effort (compare the area delimited by the dotted and continuous line for different number of pools).

#### The effect of the order of releasing enzymes on coverage and PCR effort

In simulations with MECS, we noticed that the total number of PCR reactions was affected by the order in which releasing enzymes were applied. We investigated this phenomenon systematically by simulating cDNA-AFLP for *Arabidopsis *RefSeq set (Tab. 1); in these simulations, MboI was the marking enzyme, and the releasing enzymes (TasI, ApoI, and Cfr10I) were applied in different orders. TasI is a frequent cutter, ApoI cleaves with an intermediate frequency, and Cfr10I cuts relatively infrequently (see Tab. 2). After simulating all six orders of these releasing enzymes, we found that while the coverage was similar in all simulations, the total number of PCR reactions fluctuated between 80 and 260 (data not shown).

The reason for this phenomenon was that the sizes of fragment pools differed. A large DNA pool must be partitioned into more sets (using a higher number of selective nucleotides), resulting in a higher number of PCR reactions. When all releasing enzymes cleave a cDNA set with similar frequencies, the size of fragment pools tends to decrease in pools consecutively released from the column as the amount of DNA substrate for the enzymes decreases. We hypothesized that using frequently cleaving enzymes at early steps would enhance this tendency while using less frequently cutting enzymes at the beginning would compensate for the loss of DNA substrate during sequential digestion. Simulations have not confirmed this assumption: When many enzyme combinations were compared, no correlation was found between the size of the PCR effort and the orders of enzymes cutting with different frequencies (Tab. 4).

**Table 4:** Effect of the order of releasing enzymes on cDNA-AFLP performance

**RE 1**	**RE 2**	**RE 3**	**Coverage**	**Redundancy**	**PCR samples**	**Ø Fragment length (nucleotides)**
L	M	H	65 %	38 %	713	226

L	H	M	65 %	38 %	624	219

M	L	H	65 %	38 %	672	226

M	H	L	65 %	38 %	648	226

H	L	M	64 %	38 %	552	217

H	M	L	64 %	38 %	552	217

Simulation of cDNA-AFLP with sequential digestion protocol on Arabidopsis RefSeq data was performed using marking enzymes MboI and FatI and six releasing enzymes with different frequencies of occurrence: frequently cutting enzyms H (TasI and MseI), enzymes with a medium frequency of occurrence M (BsaWI and TatI) and infrequently cutting enzymes L (AcyI and CfrI). For each permutation of H, L and M enzyme classes, cDNA-AFLP were simulated for 8 permutations of the releasing enzymes (RE). Average values of coverage, redundancy, PCR effort and fragment length for each permutation of H, L and M were calculated.

#### Minimization of redundancy by pre-restriction of immobilized cDNA

The use of more than one marking enzyme generates redundant signals for sequences that contain targets for two or more enzymes. This redundancy can be reduced by treating each immobilized DNA set with the marking enzymes used for the other sets. This treatment is called pre-restriction. All immobilized sets can eventually be digested with all marking enzymes, which we designated "complete pre-restriction." In this case, only the selection of the adapters for ligation determines which enzyme-generated DNA ends are used for marking and which merely reduce redundancy. When some immobilized sets are pre-restricted while others are not, we designate the treatment "partial pre-restriction."

Pre-restriction suppresses redundancy and thus reduces the total number of PCR reactions but it may reduce the coverage. Consider a cDNA fragment containing recognition sites for marking enzymes A (close to 5' end) and B (close to 3' end). When target sites for releasing enzymes are present between the site B and the 3' end, the fragment may be detected in both A- and B-labeled pools (depending on the length of fragments liberated by the releasing enzymes). Pre-restriction of the set to which adapter A will be ligated (called set A) with enzyme B removes site A and prevents the detection of this fragment in set A. The fragment might still be detected in set B (regardless of whether set B was pretreated with enzyme A). If, however, no target for the releasing enzymes exists between site B and 3' end, or when the distance between site B and the closest site for a releasing enzyme on its 3' side is too short, the fragment will escape detection in set B. In such a case, pre-restriction with enzyme A reduces coverage rather than redundancy. Partial pre-restriction (i.e., pre-restriction of some but not all immobilized cDNA sets) may be used as a trade-off between redundancy and coverage reduction. MECS implements optional partial pre-restriction. When pre-restriction is activated, the first marking enzyme is applied to all sets of immobilized DNA.

The effect of pretreatment on coverage, redundancy, and fragment length was investigated by simulating cDNA-AFLP on three sequence sets using two marking enzymes and three releasing enzymes. The results are shown in Tab. 5. Complete pre-restriction entirely eliminated redundancy, resulting in a significant reduction of the number of PCR reactions for all three cDNA sets. At the same time, the coverage dropped by 22–37%, and the average size of detected fragments was reduced by 22–41%. Partial pre-restriction in the form used in the simulation (one cDNA set was treated with both marking enzymes, the other was digested only with its cognate marking enzyme) led to unpredictable changes both in coverage and the number of PCR reactions, but it did not significantly affect the fragment size. Partial pre-restriction under the simulated conditions is therefore not recommended. Complete pre-restriction is advantageous when resources are limited. For example, 52% coverage can be achieved in *A. thaliana *with a complete pre-restriction. Given the same number of PCR reactions without pre-restriction, the expected coverage would be only 28% (calculated from data in Tab. 5). When resources are not limited, pre-restriction should not be used. In the *A. thaliana *case, 78% coverage can be achieved without pre-restriction for the cost of three times more PCR reactions.

**Table 5:** Pre-restriction of immobilized cDNA

	**No pre-restriction**		**Partial pre-restriction**		**Full pre-restriction**	
***Arabidopsis thaliana***						

**Marking enzymes**	FatI	MboI	HinPI	MboI	FatI	MboI

**Releasing enzymes**	MboI	FatI	MseI	MseI	MboI	FatI
	MseI	MseI	TasI	TasI	MseI	MseI
	TasI	TasI	TaqI	TaqI	TasI	TasI

**Sequence coverage**	78 %		70 %		52 %	

**Sequence redundancy**	30 %		30 %		0 %	

**Average fragment length (nt)**	183		237		142	

**PCR reactions**	100 %		125 %		36 %	

**Mouse**						

**Marking enzymes**	MboI	Csp6I	HinPI	MboI	MboI	Csp6I

**Releasing enzymes**	Csp6I	MseI	TasI	TasI	Csp6I	MseI
	MseI	MboI	MseI	MseI	MseI	MboI
	FatI	FatI	TaqI	TaqI	FatI	FatI

**Sequence coverage**	87 %		81 %		50 %	

**Sequence redundancy**	41 %		35 %		0 %	

**Average fragment length (nt)**	270		271		159	

**PCR reactions**	100 %		39 %		25 %	

**Human**						

**Marking enzymes**	MboI	Csp6I	HinPI	MboI	MboI	Csp6I

**Releasing enzymes**	CspI	MboI	MseI	MseI	CspI	MboI
	HinPI	FatI	TasI	TasI	HinPI	FatI
	FatI	HinPI	TaqI	TaqI	FatI	HinPI

**Sequence coverage**	85 %		84 %		63 %	

**Sequence redundancy**	35 %		40 %		0 %	

**Average fragment length (nt)**	274		292		182	

**PCR reactions**	100 %		86 %		61 %	

Simulation of cDNA-AFLP with sequential digestion protocol on Arabidopsis, human and mouse RefSeq ESTs was performed. Optimal combination of restriction enzymes (2 marking in combination with 3 releasing enzymes) with the highest sequence coverage was used to compare the effect of partial pre-restriction and full pre-restriction options on maximal transcript coverage, sequence redundancy, average fragment length and reduction of PCR effort (relative to the maximal number of PCR by one of the options).

### Low quality EST impede simulation

**Figure 5:**
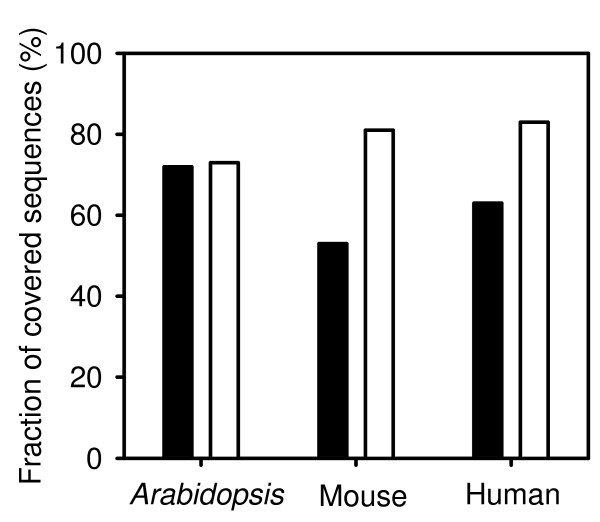
**Effect of sequence quality on coverage**. cDNA-AFLP were simulated on RefSeq and UniGene sequences using the sequential digestion protocol with two marking and three releasing enzymes. Enzyme combinations leading to the highest coverage were selected from the set listed in Tab. 2. Black bar: UniGene; white bar: RefSeq.

While comparing cDNA-AFLP protocols, we noticed that the quality of sequence data used for the simulations affected the coverage. Comparing the results obtained with RefSeq and UniGene cDNA sets confirmed this observation in that coverage was lower for the UniGene set (Fig. 5). RefSeq are subsets of high-quality sequences from UniGene (see Tab. 1 for details). The RefSeq and UniGene data sets for *A. thaliana *differ mainly in the higher number of ambiguous bases in UniGene data while the average sequence lengths are similar. Mouse and human data differ much more in the average sequence length but the difference in the proportion of ambiguous nucleotides is comparable to *A. thaliana *data.

Results in Fig. 5 indicated that average fragment length rather than quality of sequence data was responsible for the improved coverage of RefSeq sets vs. UniGene data. We investigated this phenomenon in detail by generating defined low-quality sequence data. This was achieved by introducing ambiguous nucleotides at random positions and artificially truncating EST sequences from RefSeq sets from their 5' end. When encountering a potential recognition site for a restriction enzyme that contains an ambiguous nucleotide, the software classifies the position as if no recognition sites were present. Truncated sequences may lose recognition sites for restriction enzymes, too. Both manipulations potentially decrease the coverage.

**Figure 6:**
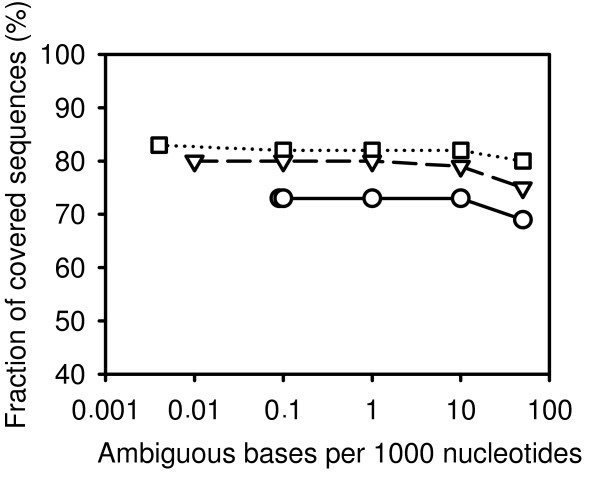
**Effect of ambiguous nucleotides on cDNA-AFLP coverage as estimated by simulation**. Modified EST sets were generated from RefSeq sequences by labeling different fractions of randomly selected nucleotides as ambiguous using a Perl script. cDNA-AFLP sequential digestion protocol was simulated using the optimal combinations of two marking and three releasing enzymes, and the coverage was plotted against the fraction of ambiguous nucleotides. Squares connected by a dotted line: mouse; triangles connected by a dashed line: human; circles connected by a filled line: *Arabidopsis*.

**Figure 7:**
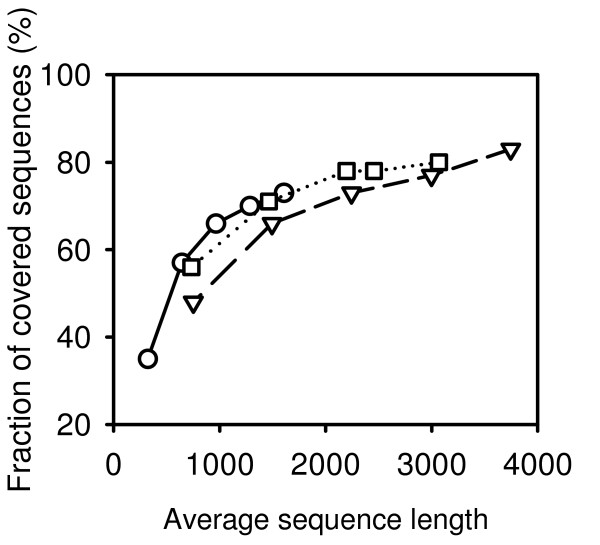
**Effect of sequence length on coverage**. Modified EST sets were generated from RefSeq sequences by truncating the sequences from the 5' end to a various extent. cDNA-AFLP sequential digestion protocol was simulated using the optimal combinations of two marking and three releasing enzymes. Squares connected by a dotted line: mouse; triangles connected by a dashed line: human; circles connected by a filled line: *Arabidopsis*.

The results of cDNA-AFLP simulations on polluted and truncated data using a sequential digestion protocol are shown in Figs. 6 and 7. The simulations confirmed that the length of EST sequences greatly affects the coverage of cDNA-AFLP. The simulation tolerates rather high levels of ambiguous nucleotides. While ambiguous nucleotides do not occur in real cDNA-AFLP experiments, the presence of short cDNA sequences is a typical consequence of RNA degradation. Our results emphasize the importance of measures increasing the length of cDNA sequences, such as protection of mRNA from degradation and the use of RNaseH-defective reverse transcriptase.

## Conclusion

Simulation of cDNA-AFLP on transcripts from *Arabidopsis thaliana*, mouse, and human revealed that sequential digestion of immobilized cDNA provides the best performance among cDNA-AFLP protocols in terms of coverage, redundancy, fragment length, and the total number of PCRs. Pre-digestion of immobilized cDNA with marking enzymes not used for marking as a redundancy-reducing measure does not improve the overall performance of the method. As a trade-off between minimizing the number of bands co-migrating during electrophoresis and maximizing PCR reactions with products suitable for normalization, primers generating 30 to 70 amplicons per reaction provide the highest fraction of analyzable fragments. While the sequential application of two marking enzymes and two to three releasing enzymes is suitable for cDNA-AFLP profiling in *A. thaliana*, human, and mice, simulations on genuine EST sequences are recommended for optimizing the cDNA-AFLP strategy for organisms with different transcriptome characteristics.

## Materials and methods

### Sequence data

EST collections of *Arabidopsis thaliana*, *Mus musculus*, and *Homo sapiens *were obtained from NCBI UniGene database [21]. The UniGene database consists of non-redundant, curated collections of transcript sequences of *Arabidopsis*, mouse, and human. In addition to full-length sequences of well-characterized genes, partial transcript sequences of at least 100 bp have been included in UniGene databases. While splicing variants for a gene are unified to a single entry, ESTs often contain non-overlapping 5' and 3' reads from the same cDNA clone, which leads to redundant representation of such transcripts. The NCBI Reference Sequence (RefSeq) database [22] consists of high-quality nonredundant sequences. Differences in data quality between these two sets are summarized in Tab. 1.

### Restriction enzymes

A collection of 18 commercially available restriction enzymes (tetra- and penta-cutters) was used for cDNA-AFLP simulation (Tab. 2). Recognition sites of these enzymes are single palindrome sequences, and cleavage generates 5'-end overhangs.

### Software

For the present study, we developed an interactive PERL script 'MECS' (multiple enzyme cDNA simulation) that simulates different cDNA-AFLP protocols for a given set of transcripts. Depending on the protocol, MECS identifies restriction sites for a given set of restriction enzymes on the input set of transcripts, generates fragment pools, and evaluates statistical features such as coverage, redundancy, and average fragment length.

## Availability and requirements

Project name: Multiple Enzyme CDNA Simulation (MECS)

Operating system(s): Any platform with Perl installed.

Programming language: Perl

Other requirements: -

License: Free

Perl code, a user guide and additional files needed for the use of the software are provided in Additional file 1 (a ZIP archive).

## List of abbreviations

AFLP: amplified fragment length polymorphism; bp: base pair; EST: expressed sequence tag; MECS: Multiple Enzyme cDNA-AFLP Simulation Software.

## Authors' contributions

AW suggested sequential digestion of immobilized DNA, performed computer simulations and wrote parts of the manuscript. DP wrote MECS tool and extended its functionality according to the needs of the project. BM supervised software development and participated on writing the manuscript. PK guided the experimental work, calculated fragment size distribution and wrote parts of the manuscript. All authors read and approved the final version of the manuscript.

## Supplementary Material

Additional file 1 **Software tool MECS (Multiple Enzyme cDNA-AFLP Simulation) written in Perl.** Click here for file
